# Assessment of the public-private-partnerships model of a national health insurance scheme in India

**DOI:** 10.1016/j.socscimed.2019.112634

**Published:** 2019-12

**Authors:** Sonalini Khetrapal, Arnab Acharya, Anne Mills

**Affiliations:** a Former PhD student, Department of Global Health and Development, London School of Hygiene and Tropical Medicine, London, UK; b Honorary Associate Professor, Department of Global Health and Development, London School of Hygiene and Tropical Medicine, London, UK; c Deputy Director & Provost and Professor of Health Economics and Policy, London School of Hygiene and Tropical Medicine, London, UK

**Keywords:** India, Health contracting, Health insurance, Public-private partnerships, PPP, RSBY

## Abstract

A single hospital admission can deplete household resources so considerably as to induce impoverishment, especially in the Indian context of low government healthcare expenditure. Rashtriya Swasthya Bima Yojana (RSBY) was a national health insurance scheme for below-poverty-line Indian families, to provide improved access to hospitalization and greater financial protection via a public-private-partnership employing private sector implementation capacity. Study objectives were to understand governance (including regulatory) environment and contract arrangements; evaluate expansion of services to beneficiaries; and assess compliance of providers and user satisfaction. A case study approach in two districts met the need for in-depth information on scheme functioning, and RSBY implementation was examined between 2011 and 13 in Patiala (Punjab) and Yamunanagar (Haryana). Methods included 20 key stakeholder interviews, analysis of secondary datasets on beneficiaries and claims, primary data collection in 31 public and private hospitals and in greater depth in 12 hospitals, and an exit survey of 751 patients. Enrolled and non-enrolled hospitals were mapped in each district and service availability of enrolled hospitals assessed; enrollee characteristics were analysed; for the 12 hospitals, information was obtained on structural quality and process of care, and patient satisfaction and out-of-pocket payments.

The Indian states and the government of India did not specify formal regulatory and implementation procedures in detail and states largely contracted out their functions to private insurance firms. Findings show regulatory weaknesses, and contractual breaches. Enrolment rates were low in both districts and more so for Patiala and there was limited access to services. There was little difference in process of care between public and private hospitals, though the structural capacity of private hospitals was better than public hospitals. RSBY helped improve accessibility and gave some degree of financial protection to patients. It also actively engaged with existing resources in the Indian health care and insurance markets.

## Highlights

• Contract specification and compliance were major challenges in RSBY.
• Out-of-pocket payment for care was significant, especially in private hospitals.
• There were few differences in care quality between public and private hospitals.
• RSBY patients were slightly more satisfied than non-RSBY patients.
• PPP schemes demand effective regulatory and monitoring systems.

## 1 Introduction

New Public Management (NPM) has been a dominant paradigm in the discipline of public administration (Arora, 2003), encouraging policies which have included market orientation of public services, contracting out, and privatization (Kalimullah et al., 2012). Public-Private-Partnerships (PPPs) have been an area of particular interest, as a new tool for supporting public service provision. The Government of India (GoI), since the economic liberalization reforms of 1980s, envisions significant untapped potential for the use of a PPP model in the health sector and has developed enabling tools and activities to encourage private sector investment and engagement (Government of India, 2011).

India's general government health expenditure to GDP ratio is about 1% (2015–16) or about 25% of total health expenditure, amongst the lowest in the world. Out-of-pocket expenditure amounts to nearly 65%, one of the highest rates globally (World Health Organization, 2018; World Bank, 2018). One-time high expenditure can deplete household resources so considerably as to induce impoverishment (NSS, 2015).

To help redress this situation, the Government of India adopted a health insurance programme called Rashtriya Swasthya Bima Yojana (RSBY) in 2008. The RSBY was a national health insurance scheme launched by the Ministry of Labour and Employment (MoLE) for below poverty line (BPL) families in the informal sector, to improve access to hospitalization and ensure greater financial protection from financial liabilities arising out of health problems that involved hospitalization. Every BPL family, on paying INR 30 annually (US$ 0.62 at 2008 exchange rate), was given a biometric-enabled smart card containing their fingerprints and photographs. The enrolment made accessible more than 700 inpatient health-care procedures to a value of INR 30000 per year per family (US$ 625).

States selected into implementing the scheme; the premium was approximately INR 800 per family, with the federal government contributing 75% and the participating state the rest. The scheme involved a multitude of stakeholders from both public and private sectors and was based on a Public-Private-Partnership (PPP) model, governed by contractual agreements. Key private stakeholders were insurance firms who bid for the contracts (specifying the value of the household premium they would require) and were responsible for scheme implementation such as empanelment, enrolment and awareness generation, and claim settlement; Third Party Administrators (TPAs) who supported insurance firms in scheme implementation - primarily in enrolment and claim settlement; and private hospitals who provided care (along with public hospitals) and claimed reimbursement according to a schedule of case-based fees.

The Indian health sector has for long had a large and vibrant private sector presence - both formal and informal. Although similar procedures in the private sector are about four times costlier than in the public sector, a majority of cases are treated in the private sector (NSS, 2015). Private insurance firms have a significant presence, with insurance regulatory authorities in 2000 framing regulation regarding registration of insurance firms and protection of policyholders' interests. Limited NGO and state health insurance has existed for some time. The choice of a PPP model and use of private as well as public hospitals in RSBY can be understood in the light of the goal to rapidly scale up coverage, making use of both the management capacity as well as the hospital capacity present in the private sector. Although many health systems make use of the private sector for delivery of care, the reliance of RSBY on insurance firm intermediaries rather than public governance structures is unusual. Thus, it is of considerable interest to examine in-depth how well RSBY's implementation mechanisms functioned given the PPP framework and the aim to increase accessibility to hospitalization and financial protection.

Literature from middle and low-income countries strongly suggests that critical influences on the success of contractual arrangements include capacities of both purchasers and providers, ability to assert control over contract compliance, and the functioning of overall governance including the regulatory framework (Mills, 1998). Moreover, literature has also noted that social health insurance in India is difficult to implement given its large informal sector, lack of cohesion and solidarity, and poor institutional capacity (Rao, 2005), and that private sector regulation is poor (Peters and Muraleedharan, 2008). Hence, the objectives of this study, conducted in 2011–12, focused on describing and analysing RSBY's governance (including regulatory) environment and contract terms; evaluating expansion of provision of care to beneficiaries; and assessing compliance of providers with the scheme and user satisfaction.

Our assessment is particularly important and timely as the PPP framework of RSBY has been adopted for Ayushman Bharat Yojana launched in 2018 and into which RSBY has been subsumed.

## 2 Materials and methods

Limited nation-wide data were available to examine RSBY contractual arrangements, and an in-depth study would not have been feasible on a large scale. Case studies of the implementation of RSBY were done in two districts, Patiala and Yamunanagar, in the adjacent States of Punjab and Haryana. These areas have both common and contrasting features, allowing for a focused analysis of the RSBY implementation process. They are near Delhi, one of the richer cities in India, and have similar culture, level of economic development, population, and state government size. The states incorporated the programme at the same time, had greater than two years of implementation at the start of the study and buy-in to the study from various stakeholders. Given that RSBY was initiated by the federal Ministry of Labour and Employment (and subsequently passed to the Ministry of Health and Family Welfare), an important contrasting feature was that governance responsibility was assigned in Punjab to the state Ministry of Health and Family Welfare (MoHFW), and in Haryana to the Ministry of Labour and Employment (MoLE).

**Fig. 1:**
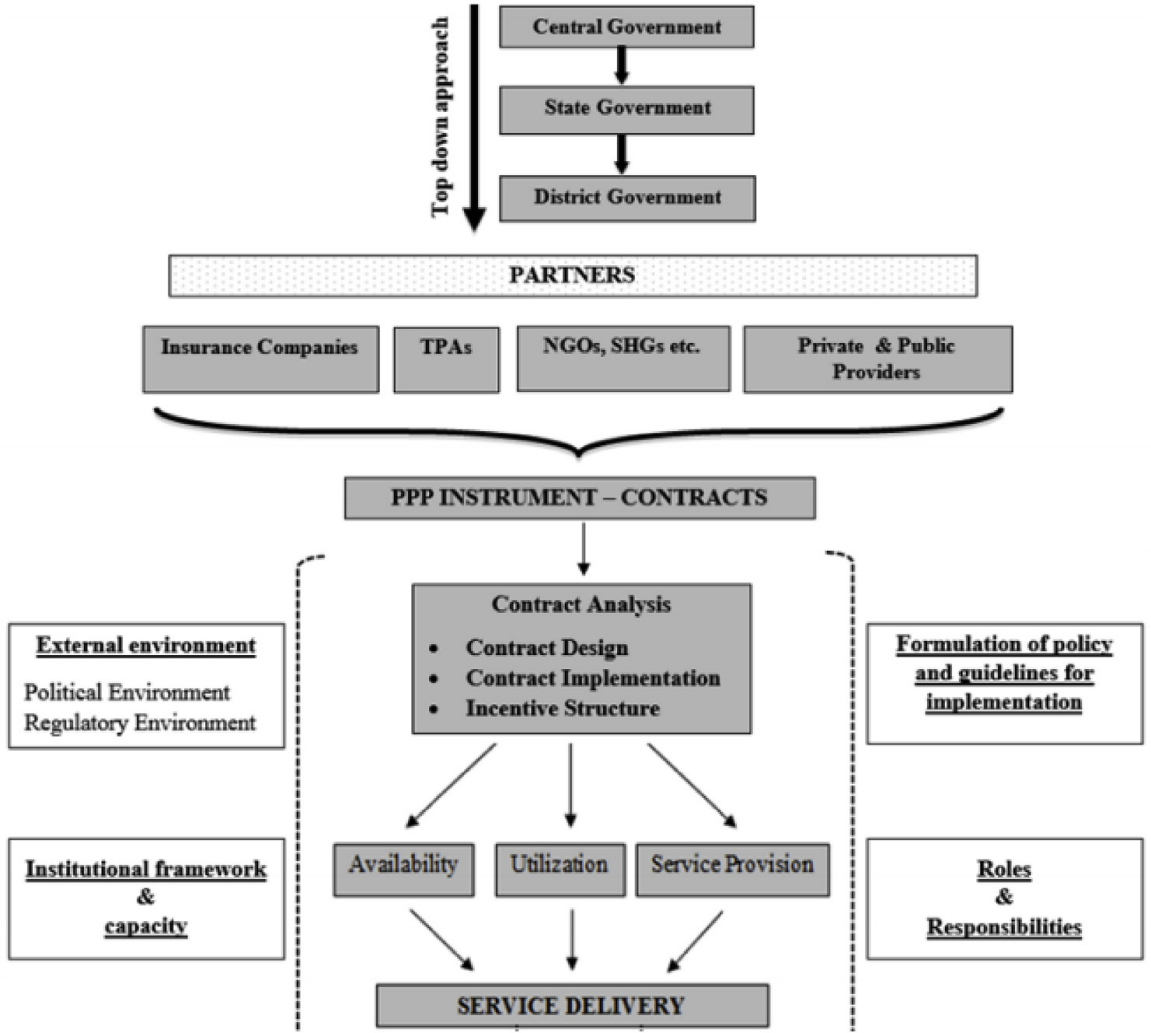
Conceptual Framework, TPA – third party administrator; NGO – non-governmental organization; SHG – self-help group; PPP – public-private partnership.

**Fig. 2:**
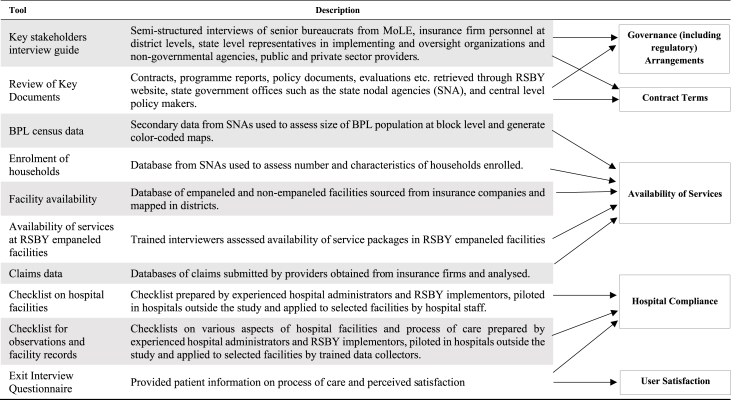
Tools used and data obtained in relation to study objectives.

Fig. 1 provides the conceptual framework and indicates that implementation of RSBY involved insurance firms, TPAs, NGOs, self-help groups (SHGs) and private and public providers with contractual relations specifying roles and responsibilities for providing services to RSBY beneficiaries. The methods and data needed for the study were identified by systematically thinking through how RSBY was intended to function given its desired aims and implementing arrangements, and - given lessons from the international and Indian literature on challenges with PPPs, contracts, and the private sector - what critical elements needed to be in place for the aims to be achieved. Elements considered critical included aspects of the environment (e.g. governance structure), design (e.g. sufficiently specified contract terms) and operation (e.g. compliance of hospitals with contractual requirements). Mixed methods involving both primary and secondary data collection were used to study governance (including regulatory) arrangements, contract terms, availability of services for the insured, hospital compliance with contract terms and service delivery, and user satisfaction with hospital care received. The study was conducted between 2011 and 2013; Fig. 2 details the tools used, and data obtained.

Semi-structured interviews of 20 key stakeholders and reviews of key documents explored governance, regulatory and contract issues. Stakeholders were identified through determining key figures that would help implement RSBY at state and district levels. Policymakers at central level were also interviewed as they had insights into how the scheme was developed. To assess competition for the contracts, the state nodal agency (SNA) of Punjab, responsible for scheme implementation, provided the list of insurance companies who had participated in the bidding process during 2008–12 (similar data were not available for Yamunanagar).

Expansion of services for the insured was assessed through secondary analysis of the size and distribution of the BPL population, data on beneficiary enrolment, a database of empaneled and non-empaneled facilities, and a claims database covering one complete annual cycle of enrolment, from September 2011 to December 2012 containing 992 claims from Patiala and 6,043 claims from Yamunanagar. ArcGIS® mapping software was used to map health facilities in Patiala and Yamunanagar. Primary data were collected by trained interviewers on the availability at 31 empaneled hospitals of 20 services reflecting categories defined under the list of package rates of RSBY.

Assessment of the quality of services followed the Donabedian Framework (Donabedian, 1988, 2005) of structure, process, outcome, and included aspects which reflected compliance with RSBY contract terms:

1. Structure: Evaluation of all structural aspects of care (e.g. facilities, equipment, staff) via a checklist completed by hospital staff.
2. Process: Evaluation of process of care via observations by trained interviewers of the functioning of the various departments and of facility records, and via information from an exit survey of patients, covering patient time spent in the clinic, adequacy of waiting area, patient privacy, etc.
3. Outcome: User satisfaction/perceived quality from the exit survey.

Impact on health outcomes was excluded from evaluation due to resource constraints and measurement difficulty.

**Fig. A1:**
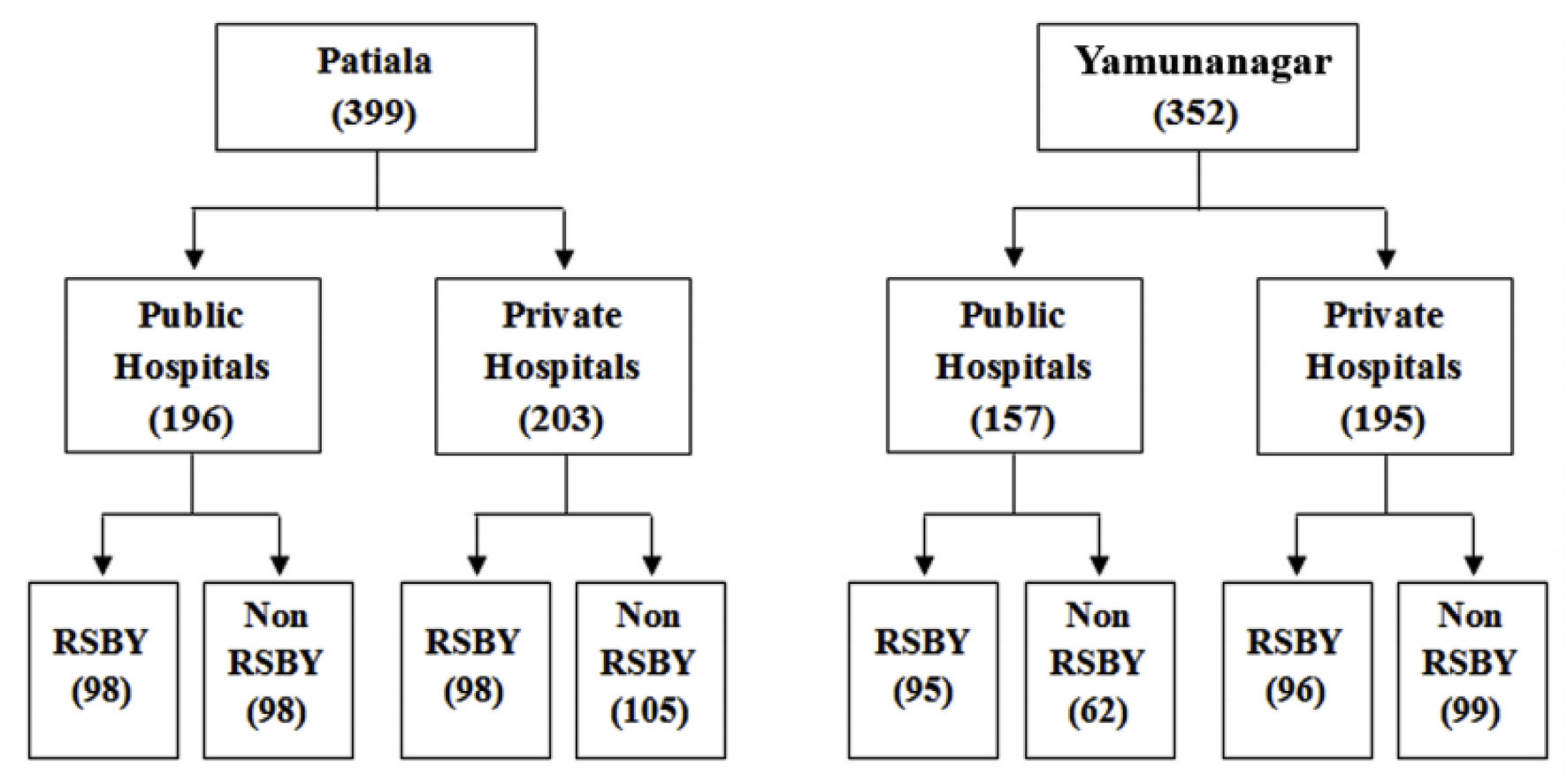
Exit interview sample size.

Twelve hospitals, three public and three private hospitals in each district, were selected for this assessment of quality of care, in order to obtain more detailed information. Factors influencing selection of the facilities were (1) the volume of RSBY patients at the facility, and (2) the management's willingness to participate. The exit survey used a semi-structured, pre-tested questionnaire and trained interviewers to obtain information from both RSBY and non-RSBY patients (in order to explore differences between them) at discharge from hospital or during follow-up visits. The intended sample size was 752, based on a case control study design, with 95% confidence interval, 80% power, 20% expected frequency of exposure (scheme implementation) in the control group and 10% exposure among the cases. As patients left the selected empaneled hospitals they were interviewed until the desired sample size was reached in each facility and each group (i.e. RSBY or non-RSBY). Exclusion criteria were enrolment in any other insurance scheme, and patient refusal. 751 exit interviews were completed (Figure A1)

Questions covered patient characteristics, service delivery (indicators of process and quality of care) and user satisfaction. Service delivery included key aspects related to hospital compliance: RSBY help desk; coverage of cost of treatment; diagnostics and medicines provision; provision of food and transportation services; post hospitalization cover. To assess user satisfaction, a modified version was used of the questions in the Hospital Consumer Assessment of Healthcare Providers and Systems (HCAHPS, 2012) questionnaire. Questions covered seven categories: care during admission, care from nursing staff, care from doctors, hospital environment, experiences in the hospital, care during discharge and overall rating by beneficiaries.

The qualitative data from interviews and document review were analysed using thematic analysis (Braun and Clarke, 2006) and triangulated where possible. The analysis involved recognizing key themes from documents and key-informant interviews. Quantitative data were initially analysed using descriptive statistics and multivariate analysis was undertaken when appropriate and feasible.

Ethical clearance was obtained from the Institutional Ethics Committee at the London School of Hygiene (5968) and Tropical Medicine and the Public Health Foundation of India (TRC-IEC-133/12).

## 3 Results

The governance framework driving the PPP arrangement was expected to affect the enrolment of eligible beneficiaries, the availability and accessibility of the providers who were contracted to treat them, and the services received by users. The results are thus organized around four themes considered critical in influencing Scheme operation: (i) governance arrangements including regulatory mechanisms and contractual framework (ii) contract terms (iii) expansion of services through enrolment of beneficiaries and empanelment of service providers within the PPP framework and (iv) care received within the contractual framework and the beneficiary perception of care.

### 3.1 Governance including regulatory arrangements

The governance framework is made up of the institutions which enforce contracts with administrative implementers who find the enrollees, locate providers, make arrangements with providers and ensure ongoing quality control. The arrangements are shaped by the general political and regulatory framework within which most public programmes work.

Key informants and document review indicated that RSBY was announced a few months before the 2009 general elections by a government eager to be seen as pro-poor and suggested that the scheme content and implementation process may not have been well thought through. Lessons in relation to regulation, implementation, monitoring, etc. from similar insurance schemes within India such as the Universal Health Insurance Scheme, and from other countries, were not incorporated in RSBY (Rao, 2005). As public healthcare delivery is a state responsibility in India, the design of RSBY required political commitment at the state level; which party was in power at the State level did not affect scheme implementation.

Consistent with findings from other research and document reviews, key informants indicated that the regulatory framework was weak, particularly for private health facilities. Regulation of private facilities was very limited and most likely went unenforced (Gupta and Rani, 2004; Peters and Muraleedharan, 2008). Despite a substantial presence of private hospitals in the country, information regarding their number, structure, functioning, type and quality of care was grossly inadequate (Panning Commission, 2007). The Clinical Establishments Act was passed in 2010 and notified by the union government in 2012, three years after RSBY was introduced. All states were asked by the Ministry of Health and Family Welfare (MoHFW) to adopt the law but only 9 of 29 states had done so by the end of 2014. The Indian Insurance Regulatory and Development Authority (IRDA) was the regulating body for insurance companies and the TPAs. IRDA guidelines for insurance companies and TPAs were mandatory, and compliance required obtaining a license to provide health insurance; hence insurance regulation, unlike health provider regulation, was enforced.

**Fig. 3:**
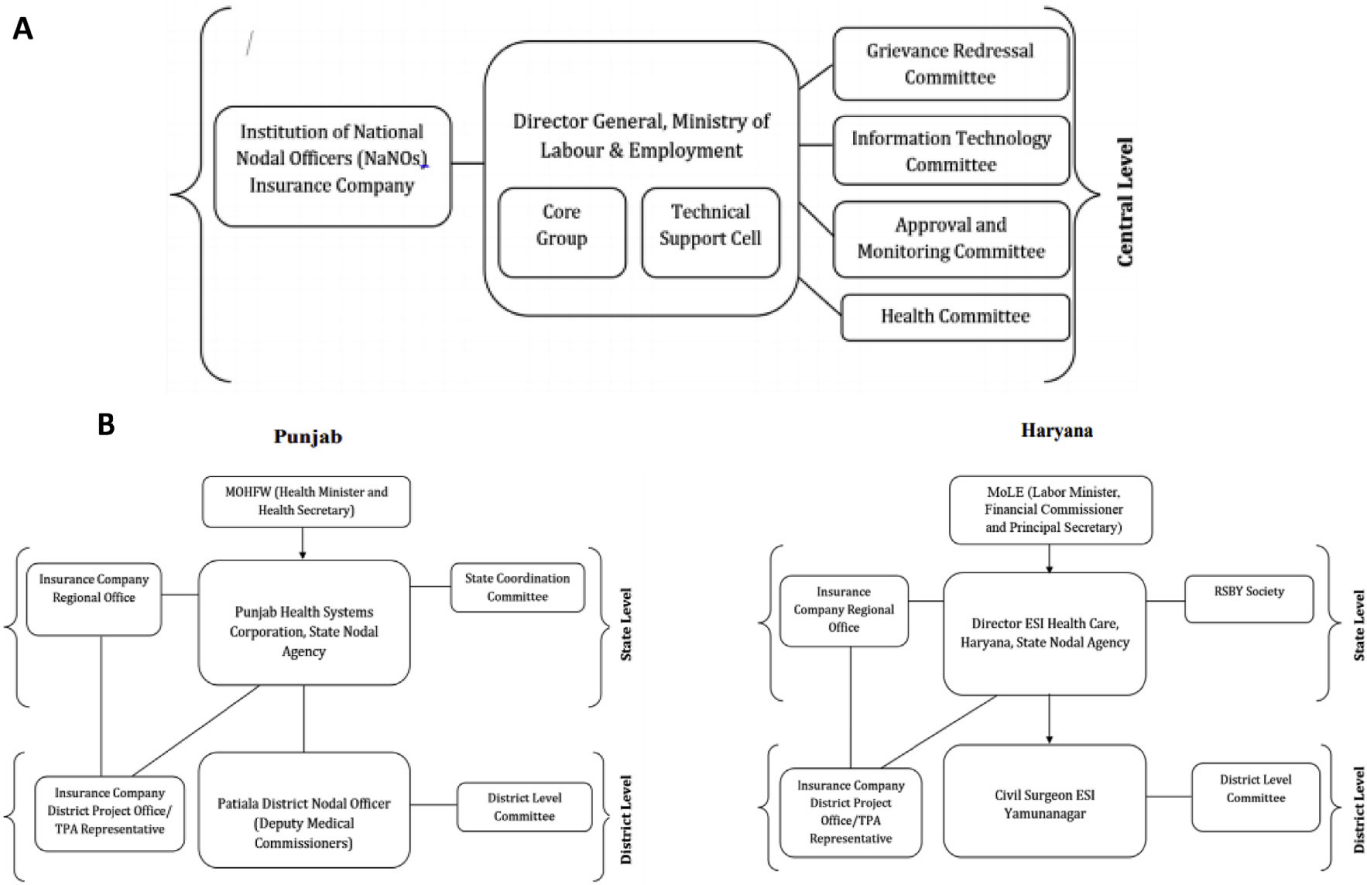
Central (A) and State (B) level Structures.

No formal organizational structure supporting RSBY was specified, and the actual arrangements at central level and in the two states are shown in Fig. 3. No new bureaucracy was created to implement RSBY and the central team was small. The existing structure of the Directorate General Labour Welfare rolled out the scheme using the available government functionaries who were assigned additional responsibilities. RSBY was implemented by the Ministry of Labour and Employment, which was considered to lack the experience to implement a health scheme of such complexity. Different state governments chose different departments for placement of the State Nodal Agencies which took responsibility for scheme implementation (Fig. 3 B). Punjab chose the Health Systems Corporation (part of the Department of Health and Family Welfare, Government of Punjab) and Haryana chose the Directorate of Employees State Insurance Health Care within the Ministry of Labour and Employment, Government of Haryana. These influenced the nodal agencies designated at the district level: in Patiala it was the Deputy Medical Commissioners and in Yamunanagar it was the civil surgeon who was both the state nodal officer and also responsible for the four adjoining districts. In general, key informants considered human resources to be inadequate for implementation at state and district levels. For example, no single position was dedicated to RSBY at the state level.

Out of eight insurance companies who bid in Punjab for the RSBY contract, ICICI Lombard General Insurance Company was selected. Such bidding information was not available for Haryana, but the same insurance company (ICICI) held the contract. The insurance company had contracted one TPA in both districts to facilitate RSBY implementation. This contract award was based on the TPA's reputation with no bidding process.

Private and public providers were contracted to deliver services to RSBY beneficiaries. In addition, other partners such as NGOs and self-help groups assisted the insurance company in raising awareness of the scheme. The majority of scheme implementation activities were undertaken by insurance firms, with state governments mainly playing a facilitating role.

### 3.2 Contract terms

Contracts under RSBY were standardized and all states used the same contract. Important contracts were at three levels, between: (i) the centre and state; (ii) the state and insurance company; and (iii) the insurance company and service provider (Table 1). The contractual agreement between the Central Government and the states was identical for Haryana and Punjab. Although there was room for innovation and additional clauses in the Central Government's RSBY framework, the states under study had not exercised that flexibility. The purpose of the contract was clearly outlined to provide social security to the BPL workers and their families in the unorganized sector. The contract clearly defines the roles and responsibilities of both parties involved, the Central Government and the SNA.

**Table 1:** Contract terms.

Contract terms	Contracts between		
	Central & state government	State & insurance company	Insurance company & provider
Ownership of contracts stated (signing authority)	yes (Director General Labor Welfare, Ministry of Labor and Employment, Government of India and the respective state government)	yes (Department of Health & Family Welfare, Government of Punjab through State nodal agency and ICICI Lombard General Insurance company)	yes (ICICI Lombard General Insurance company and empaneled hospitals)
Objectives of contract stated (to provide social security to the BPL workers and their families in the unorganized sector)	yes	yes	yes
Length (duration) of the contract stated	none	yes (one year)	yes (one year)
Payment mechanism specified	yes (75% by central government and 25% by the state government)	yes (electronically according to 64VB or IRDA act)	yes (electronically)
Roles & responsibilities of stakeholders stated	yes (Central government and state government specific roles were defined)	yes (State nodal agency and Insurance company roles were clearly defined)	yes (Roles of insurance company and Health care provider were defined in the contract)
Empanelment criteria of providers specified	none	yes (empanelment criteria or the healthcare provider was stated)	NA
Statement of monitoring mechanisms for contract implementation	none	none	none
Specification of sanctions	none (not expected in a federal system)	yes (contract could be terminated)	yes (contract could be terminated)
Explicit incentives for effective scheme implementation	none (not expected in a federal system)	none	none

The contract between the state government and the insurance company was the most comprehensive RSBY contract with details of contract commencement, duration and termination clearly specified. This contract was more detailed probably because the insurance company bore extensive responsibilities for scheme implementation.

A major gap in contract design was the monitoring strategy, which was loosely stated in the contract and monitoring mechanisms and parameters were not defined. There was no mention of resources required for monitoring and supervision at the district level. Further, there was no mention of incentives for stakeholders to ensure effective implementation and offer high quality of care.

### 3.3 Expansion of services

The PPP framework of RSBY was intended to enhance supply through bringing in providers who previously would have been beyond the economic reach of the beneficiaries. RSBY became the third-party-payer through enrolling beneficiaries and giving them access to providers willing to provide specified packages of care at the lower-end of market prices, specified as payment for procedures, yet nonetheless at some acceptable level of care. Insurance firms were contracted to enroll the beneficiaries and were responsible for empaneling service providers to make care accessible.

Only 15% of the eligible (BPL) population was enrolled in the RSBY scheme in Patiala district compared to 40% in Yamunanagar. Issues such as errors in the list of eligible households and the annual process of re-enrolment by the insurance companies are likely general explanations for low enrolment rates. A specific reason for very low enrolment in Patiala district, provided by a key informant, was that enrolment overlapped the harvesting season which would have occupied many potential beneficiaries, whereas enrolment in Yamunanagar was done earlier.

The maximum number of individuals that could be enrolled per family was five, but the average number enrolled per family was only 3.25 (Patiala 2.37, Yamunanagar 3.56) although both states had average household sizes of around 5.2. Low enrolment per family may indicate moral hazard as the greater the household enrolment, the higher the likely claims pay-out per family. Only in Patiala were age-gender specific enrolment rates available (Table A1). A greater percentage of those of working age (15–64) were enrolled than other age brackets with little gender difference. However, a slightly higher proportion of men than women above 64 were enrolled: 31.9% and 22.4%. Very few young people were enrolled, perhaps reflecting the fact that various other health programmes are available for the young.

**Fig. 4:**
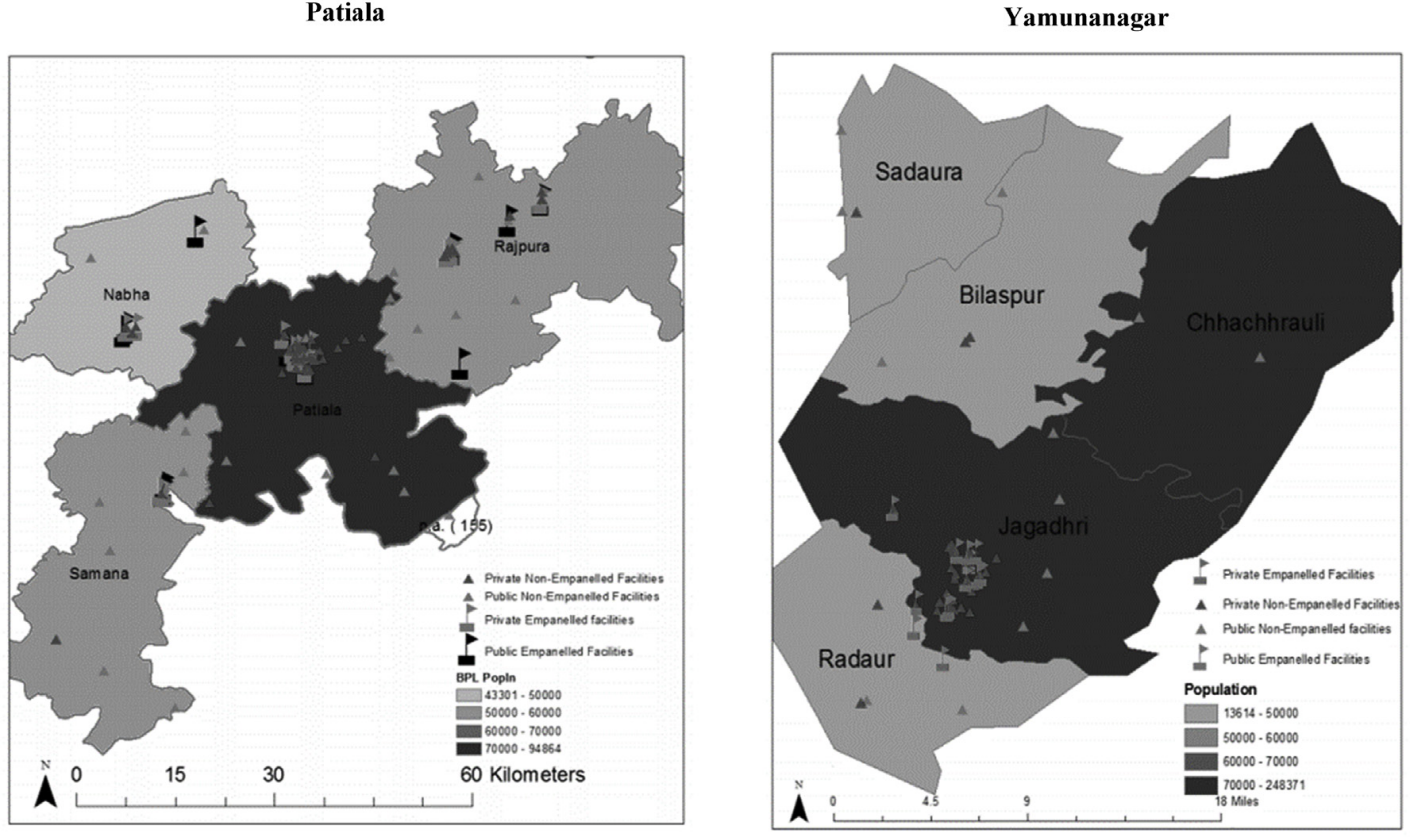
Distribution of health facilities in Patiala (4a) and Yamunanagar (4b).

Contracts require insurance firms to enlist service providers who would be available to the enrollees. The empanelment process was based on prescribed criteria that the service providers need to possess, such as specified basic facilities. For example, the service provider was required to have at least 10 inpatient medical beds, and specified medical, surgical and diagnostic facilities. The providers were reimbursed the fixed package rates for the services offered to the RSBY beneficiaries through electronic transfer by the insurance companies. In Patiala, the 10 empaneled public hospitals were distributed across the state whereas the 7 empaneled private hospitals were geographically clustered around pockets within the sub-district level (Fig. 4a). In Yamunanagar, very few public hospitals were empaneled (4); the 33 empaneled private sector hospitals were clustered around one sub-district (Fig. 4b). There were a number of hospitals, both public and private, dispersed around the districts that were not empaneled. Public hospitals shown in Fig. 4 comprise Community Health Centres, and sub-divisional and civil hospitals, which can be assumed to have the facilities required for empaneling, but the same assumption cannot be made for non-empaneled private hospitals.

An effective PPP arrangement should ensure that the entire package of care as promised through RSBY should be accessible to the enrollees. This was assessed through employing a checklist of service availability. It was completed by only 12 of 17 empaneled hospitals in Patiala, and 19 of 37 in Yamunanagar (Table 2) due to refusals, consistent with the reluctance of private hospitals to supply information to public authorities referred to earlier. The services shown in the table are the broad categories of all the packages under the RSBY scheme. Super-specialty services such as cardiology, neurology, neurosurgery and urology were minimal in RSBY-empaneled hospitals in both districts, and where present, were mainly in the private hospitals. A few private hospitals in Yamunanagar provided all services but no private hospitals did so in Patiala. None of the public hospitals in either district provided all the services covered by RSBY. Public hospitals in Patiala had only 50% of potential services actually available, and Yamunanagar 78%; these percentages were 63% and 64% for private hospitals, respectively. Given missing data, these findings are indicative of limited service availability rather than conclusive, though if anything they may overestimate service availability since smaller facilities are more likely to have been omitted.

**Table 2:** Availability of services within RSBY empaneled hospitals.

	Patiala			Yamunanagar		
	Public	Private	Total	Public	Private	Total
Number of empaneled hospitals	10	7	17	4	33	37
Number of hospitals which returned the survey	5	7	12	2	17	19
Total No. of beds available	300	146	446	NA	205 (8)^a^	NA
Mean No. of beds per hospital	60	21	37	NA	25.6	NA
**Number of hospitals with the following services:**						
Neonatal care	4	6	10	2	15	17
Burns	3	3	6	2	6	8
Snake bite	3	3	6	2	12	14
Oncology	0	3	3	2	4	6
Urology	1	6	7	0	9	9
Endocrinology	0	2	2	0	13	13
Paediatrics	4	6	10	1	9	10
Orthopaedics	4	4	8	2	9	11
Ophthalmology	4	2	6	2	15	17
Neurosurgery	0	1	1	1	8	9
Hysteroscopy	0	4	4	0	7	7
Endoscopic procedures	0	6	6	1	12	13
Gynaecology	5	6	11	2	13	15
General surgery	5	7	12	2	12	14
ENT	3	3	6	2	8	10
Dental	4	2	6	2	9	11
Medical general ward – ICU	0	5	5	2	12	14
Medical general ward –nonsurgical	5	7	12	2	14	16
Medical general ward – surgical	5	7	12	2	16	18
Intensive care unit	0	5	5	2	13	15
Total possible types of care (number of hospitals times the number of required types of services)	100	140	240	40	340	380
Actual total care (total number of services available)	50	88	138	31	216	247
Percentage of services actually available	50%	63%	58%	78%	64%	65%

a Only eight hospitals reported the number of beds available.

Information from the health provider checklist for the 12 hospitals studied in detail showed that private hospitals scored better than public hospitals under all categories of structural quality (Khetrapal, 2016). This was confirmed by the Observation and Facility Record Checklist.

Insurance claims indicate how RSBY was used to access care (Table 3). The number of claims per 1000 individuals enrolled under the scheme, for the period of 14 months, was approximately 26 for Patiala and 36 for Yamunanagar. In both districts, more claims per hospital were made in private hospitals, suggesting that the private hospitals may have been preferred or that public hospitals may not have been fully engaged with RSBY, but there was no benchmark against which the volume of claims could be assessed. Claims were clustered in certain hospitals (measured through the Herfindahl Index, calculated separately for private and public hospitals): in Patiala the claims were significantly concentrated in certain private and public hospitals; in Yamunanagar, in public hospitals only (Table 3).

**Table 3:** **Claims in Patiala and Yamunanagar (**for a 14-month period).

	Patiala	Yamunanagar
Claims in 14 months	992	6043
Claims/month	70.9	431.6
Claims/facility/month	4.2	11.7
Claims/1000 population/month	1.85	2.6

Claims distribution in private and public hospitals				

District	Type of hospital	No. of claims (%)	No. of empaneled hospitals	Average claims per hospital *(Claims/total No. of empaneled hospitals)*

Patiala	Private	669 (67.4)	7	95.6
	Public	323 (32.6)	10	32.3
	Total	**992 (100)**	**17**	**58.4**
Yamunanagar	Private	5,658 (93.6)	33	171.5
	Public	385 (6.4)	4	96.3
	Total	**6,043 (100)**	**37**	**163.3**
**Herfindahl-Hirschman Index**^a^**of hospitals**				

		**Private hospitals**	**Public hospitals**	**Total**

Patiala		3,058	3,468	1,758
Yamunanagar		1,078	5,222	967

a Interpretation: Below 1500 – un-concentrated; 1500–2500 – moderately concentrated; above 2500 – highly concentrated.

The information in Fig. 4 and Table 2 on hospital distribution and service availability suggests that not everyone would have been in easy reach of an empaneled hospital and only a limited set of services were often available, implying that not all empaneled hospitals were equally desirable. The PPP design, although drawing on private hospital capacity, was unlikely to have been able to reduce fully travel distances and the associated costs.

There was a large difference in mean claimed amount and reimbursed amount in Patiala (INR 4,134 or US$ 63) and virtually no difference in Yamunanagar (Table A2). In Patiala, mean claimed amounts were fairly similar between public and private hospitals, but a higher share of public hospital claims was reimbursed, resulting in a higher mean reimbursement to public than private hospitals. Mean claimed and reimbursed amounts in Yamunanagar were virtually identical across public and private hospitals. It is possible that the difference in the responsible state body for RSBY between the districts may have influenced this pattern: the ministry of health in Patiala might have been more restrictive in adjudicating claims than the ministry of labour in Yamunanagar. The actual mean amount paid per claim was fairly similar across public hospitals in Patiala and both public and private hospitals in Yamunanagar, with private hospitals in Patiala receiving a lower mean amount.

In both districts, most of the claims were made under the medically managed disease (MMD) general package, followed by the ophthalmology package and MMD-ICU (Table 4). Almost all the claims categorized under ophthalmology and MMD-ICU were from private hospitals. Claims under cancer ailments, endocrine and neurosurgery were almost negligible in both districts, although they are among the leading causes of hospitalization in India (NSS, 2015). There were very few paediatric cases.

**Table 4:** Distribution of claims in both districts by service type.

Disease category	Districts		Hospital type (Both Districts)	
	Patiala n = 992	Yamunanagar n = 6043	Private n = 6327	Public n = 708
	%	%	%	%
No package listed	0.1	4.6	4.3	0.8
Dental	0.1	0.1	0.1	0.1
Ear	1.2	0.1	0.1	1.8
Endocrine	0.2	0.1	0.2	0.1
Endoscopic procedures	0.9	2.2	2.1	1.1
General surgery	11.8	11.1	10.2	20.1
Gynaecology	11.5	6.3	6.4	12.4
Hysteroscopy	0.1	0.2	0.2	0.0
MMD-general	44.9	31.7	32.1	46.6
MMD-ICU	19.3	14.0	16.3	0.0
Neurosurgery	0.0	0.1	0.1	0.0
Nose	0.9	0.2	0.1	1.8
Oncology	0.1	0.0	0.0	0.0
Ophthalmology	3.4	18.9	18.1	4.8
Orthopaedic	4.3	7.0	6.5	8.2
Paediatric	0.0	0.1	0.1	0.0
Throat	0.0	0.1	0.1	0.1
Urology	1.2	3.2	3.1	1.8
**Total**	100.0	100.0	100.0	100.0

MMD: medically-managed disease; ICU: Intensive Care Unit.

### 3.4 Compliance of providers and user satisfaction

Monitoring of service delivery (process quality of care) and incentives that encourage appropriate effort are a crucial part of the success of any health scheme. PPP in health services is intended to leverage market forces that may help ensure better services. Compliance with the service contracts is strongly connected to what should be expected from service providers and satisfaction with services provided. Moreover, evidence on compliance of providers with contract terms can indicate failure of monitoring.

Hospital compliance with contract requirements was assessed largely from the exit interviews through questions about the process of service delivery (Table 5). Contract clauses specifically require, for example, provision of food, medicines and diagnostic tests during the hospital stay, reimbursement of transport costs, and coverage of post-hospitalization services for five days. Variability in compliance was observed for the various service delivery categories studied, though Patiala seemed to perform better in most of these categories than Yamunanagar. For information provided to patients from the RSBY help desk, Patiala was significantly better than Yamunanagar. Patiala also performed better in terms of providing diagnostics and medicines, since a smaller proportion of participants from Patiala were asked to obtain them from outside the hospital. Neither district did well in providing food to patients. Patiala performed better in terms of informing patients on the balance of money left on the card, providing information about post-hospitalization expenses, and reimbursing the transportation cost in Patiala (whereas no patients reported being reimbursed in Yamunanagar).

**Table 5:** Patient reports of Service Delivery.

		Districts		Hospital type (Both Districts)	
		Patiala (n = 195)	Yamunanagar (n = 191)	Private (n = 193)	Public (n = 193)
		%	%	%	%
RSBY Help desk	Separate RSBY help desk (Yes)	42.1	2.1	28.5	16.1
	Staff at RSBY help desk were helpful and polite (Yes)	95.4	97.4	95.9	96.9
Waiting Period (<15 Min)		71.3	85.3	82.4	74.1
Process of Registration	Fingerprint scanner used for fingerprint verification (Yes)	97.4	97.4	97.4	97.4
Information received from RSBY help desk	Information about treatment cost given to the patient (Yes)	48.2	8.4	24.9	32.1
	Patient was informed about the money left in the smart card	41.0	10.5	22.3	29.5
	Patient was informed about insufficient money in the card (Yes)	20.0	0.0	21.7	12.0
Diagnostics and medicines (OOP)	Patient asked to get diagnostic test from outside the hospital (Yes)	13.8	21.5	21.8	13.5
	Patient asked to get the medicines from outside the hospital (Yes)	7.7	16.2	15.5	8.3
Food provided to patients during hospital stay		20.5	36.1	34.7	21.8
Process followed during discharge	Discharge summary given to patient at the time of discharge (Yes)	84.1	100.0	89.6	94.3
	Fingerprint verification at time of discharge (Yes)	94.4	97.9	96.9	95.3
	Patient informed about balance amount in card at discharge (Yes)	47.7	20.4	33.9	34.4
Transportation cost reimbursed by hospital		45.1	0	18.1	27.5
Post-hospitalization knowledge & expenses	Knew about 5-day post- hospitalization expenses (Yes)	30.3	6.8	19.2	18.1
	Medicines were provided by the hospital (Yes)	86.9	100.0	89.4	97.3
	Diagnostic test was done free of cost (Yes)	11.1	0	0.0	11.1

With regard to public and private hospitals, only slight differences were observed for most categories, and compliance problems seemed mainly to be common across both types of hospital. Private hospitals were more likely than public hospitals to ask patients to get diagnostic tests and medicines from outside the hospital, and less likely to reimburse transport costs.

Indeed, out-of-pocket expenditure from patients in private hospitals was almost double that in public hospitals, both within each district and across the districts (Khetrapal and Acharya, 2019). It was expected that expenditures of RSBY beneficiaries would be significantly less than those of non-RSBY members. This was the case in Patiala, however in Yamunanagar, the difference was less pronounced. Insurance did not, therefore, produce a large reduction in the actual cost borne by the intended beneficiaries, in large part because of the preference for using private hospitals.

Table 6 shows information on user satisfaction, by district, type of hospital and RSBY and non-RSBY members. There was generally a high level of satisfaction across all categories, as commonly reported in exit surveys. While there were variations within and between aspects of care, user satisfaction relating to care from nurses and doctors was better among Patiala respondents when compared to Yamunanagar, and the overall hospital rating was higher. Almost all aspects were rated somewhat better in private facilities when compared to public facilities, and the overall hospital rating was slightly higher for the former. User satisfaction of RSBY participants was slightly higher on all aspects than that of non-RSBY participants, though the latter's hospital rating was slightly higher. Virtually all patients would recommend their hospital to friends, regardless of its location or type.

**Table 6:** User satisfaction.

		Patiala (n = 399)	Yamun-anagar (n = 351)	Private (n = 397)	Public (n = 353)	RSBY (n = 386)	Non RSBY (n = 364)
		%	%	%	%	%	%
Experiences during admission	Bed made available at time of admission (Yes)	98.2	98.0	97.0	99.4	99.0	97.3
	Availability of wheelchair (Yes)	96.8	100	98.8	96.8	100	97.1
	Hospital staff pushed wheelchair (Yes)	88.2	64.8	82.1	76.2	83.7	77.9
	Time taken by nursing staff (<30 min)	77.2	96.6	90.4	81.6	88.1	84.3
	Time taken by doctors (<30 min)	68.7	83.5	79.8	70.8	78.2	72.8
Care from nurses	Nurses treat patients with courtesy and respect (Yes)	85.2	80.3	86.6	78.8	86.5	79.1
	Nurses listen carefully to the patients (Yes)	87.5	83.2	89.7	80.7	87.6	83.2
	Nurses explain things in a way that patients could understand	86.2	75.5	85.9	75.9	81.9	80.5
	Patient get help as soon as he/she wanted it (Yes)	83.6	54.8	75.4	67.2	67.8	75.4
Care from doctors	Doctors treat the patients with courtesy and respect (Yes)	86.2	86.9	90.7	81.9	88.1	84.9
	Doctors listen carefully to the patients (Yes)	87.5	82.1	90.7	78.5	85.8	84.1
	Doctors explain things in a way patient could understand (Yes)	85.5	72.9	84.9	73.7	80.3	78.8
Hospital environment	Patients' surroundings & bathroom area kept clean (Yes)	80.7	85.2	92.7	71.7	85	80.5
	Patients' beds were found quiet at night (Yes)	78.2	86.3	90.4	72.5	83.2	80.8
Experiences in Hospital	Patients get help to go to the bathroom or use a bedpan (Yes)	72.5	70.7	78.6	63	72.8	71.2
	Hospital staff help to reduce the patient's pain (Yes)	89.5	89.9	92.9	85.9	91.2	88.2
	Hospital staff explain about the medicine and its side effects	71.9	24.2	62.2	47.3	55.4	54.9
Discharge Experience	Staff enquire from the patient if any help was required (Yes)	99.2	96.3	99.2	96.3	98.4	97.3
	Suggestion for any follow-up (Yes)	98.7	93.2	97.2	94.9	97.4	94.8
Hospital Rating (out of 10)^a^		7.9	5.5	6.9	6.7	6.5	7.1
Recommend Hospital to Friends		98.7	98.0	98.7	98	99.2	97.5

a Patients were asked to rate the hospital on a scale of 1–10.1 was considered very poor and 10 was excellent.

## 4 Discussion

Understanding the consequences of contractual relationships is challenging (Loevinsohn and Harding, 2005), especially in the health care sector where quality of care is difficult to assess. This study drew on a mix of methods using primary and secondary data to shed light on the operation of a PPP. However, data were incomplete regarding the characteristics of the BPL population in Yamunanagar, and availability of services for 23 RSBY empaneled hospitals who did not respond.

The study found that the scheme had been launched in a rush due to the upcoming general elections in 2009; document review and key informants suggested that this is consistent with findings that such schemes are usually announced during the election time for political gains (Thakur, 2015). Announcing or increasing fiscal expenditure for social programmes for political ends at election cycles is well documented (Ebeke and Dölcer, 2013).

Most PPP literature emphasizes that governments must play a large regulatory role if health care is to be delivered via private firms and providers (Baru, 2013; Hart, 2016) and that strong regulatory, managerial and information capacity is needed (Regional Committee for Europe – WHO, 2002). Implementation of a PPP framework requires significant government effort due to the need for strong regulation. (Regional Training Institute, 2014).

Findings show regulatory weaknesses, including significant contractual breaches such as lack of information given to beneficiaries about packages and location of hospitals, non-reimbursement of transportation cost, unavailability of food, and providers not sharing information with beneficiaries. Neither states nor the central government laid down specific implementation and regulatory procedures in states. The capitation payment per household seems to have been awarded automatically to the state then subsequently to the insurance firms, without much subsequent monitoring. Similar findings have also been observed in other studies on RSBY (Das and Leino, 2011; RSBY Committee, 2014; Seshadri et al., 2011). A stronger regulatory system may have questioned, for example, the low enrolment rate per family. The principal, GoI, designed the institutional framework, however health is a state subject thus strong state capacity was key to smooth implementation, and state governments should serve as the primary custodian and stakeholder of the scheme. Instead, the states had largely contracted out their functions to private insurance firms without much attention to designing incentives that would ensure they operated in the public good.

The study provided the opportunity to explore the implications of different governance structures at state level. Results suggest that the Department of Health in Punjab was better in terms of service delivery, transportation reimbursement, higher empanelment of public facilities and overall user satisfaction, which was 7.9 (out of 10) in Patiala, and 5.5 in Yamunanagar (Table 6). However, the Department of Labour and Employment in Haryana achieved higher enrolment of beneficiaries, greater empanelment of hospitals, and a higher rate of use of hospitals (as assessed though the claims rate, Table 3). Key stakeholders interviewed felt that it was easier to work with the Health Department than the Labour Department, and the latter may have lacked the expertise to deliver health services to the BPL population as health was not their prime responsibility.

Enrolment rates were low in both districts and more so for Patiala as compared to Yamunanagar, both in terms of percentage of households covered and those enrolled within a household, and this finding is consistent with studies of voluntary enrolment in other settings, for example Vietnam (Wagstaff, 2007). Other studies have raised concerns regarding the quality of information, education and communication (IEC) activities undertaken to promote RSBY (Trivedi and Saxena, 2013; Mahadevia, 2012). Given the poor regulatory and monitoring mechanisms, a low level of IEC should be expected from the agent. Principal agent theory suggests that agents in a PPP relationship will reduce costs (with implications for quality) when payments are based on per unit payment (Hart, 2016).

As suggested above, the difference in enrolment rates between the districts might be related to their different regulatory environments. The MoHFW (in Patiala) may not have been as efficient as the MoLE (in Yamunanagar) in identifying and enrolling those in the informal sector. Adverse selection was not evident in the scheme at least in terms of age-group enrolled, as the enrolment rate was higher for those above 25 years and poor for those below 15 years. Adverse selection is less likely in social health insurance schemes when there are no significant enrolment charges for the eligible group (Belli, 2001).

Although RSBY was intended to increase access to services for the poor, the distribution of empaneled hospitals indicated that there was likely to have been inadequate access to services in some areas. Literature shows that non-availability of RSBY empaneled hospitals has meant lower scheme utilization in some areas (Health Inc Consortium, 2014). In this study, public hospitals were more equitably distributed throughout the districts than private facilities, which were geographically clustered around pockets at the sub-district level, and not all public hospitals were empaneled. Such an observation raises questions on the empanelment criteria of the scheme. Similar findings have been reported from other states such as Karnataka (Rajasekhar et al., 2011). There could be several reasons for not empaneling available hospitals. Misalignment of incentives might be a plausible reason where insurance companies empaneled fewer hospitals in order to limit accessibility and thus minimize claims to increase their profit. Other studies have shown that insurance companies have tried to suspend or de-empanel hospitals for small infractions, and even for unintentional mistakes (Khurana and Dave, 2016). Administrative staff might also be poorly informed and not proactive, as was observed in Chhattisgarh (Council Of Tribal And Rural Development, 2013). However, it needs to be recognised that the number of hospitals in rural areas is small, and these hospitals may not all meet the eligibility criteria of empanelment; hence it is likely that the majority of empaneled hospitals will be in urban areas. The result is that rural populations have to travel long distances to access services.

The majority of claims were for private care. A preference for using private care (as noted above), previously unaffordable, probably reflects the general notion in India that the private sector offers better care. Further, clustering was seen (beneficiaries going more to certain hospitals) in both districts. Certain hospitals may develop a reputation over time because of the quality of services they provide. The scheme has enabled the poor to choose a hospital based on their preference which may have resulted in many beneficiaries choosing the most reputed hospital of the district. Under a PPP arrangement there is no built-in mechanism other than patient demand or choice that would induce a greater number of hospitals to offer more services.

In Patiala, there was a greater difference between claimed and reimbursed amounts than in Yamunanagar. Further, this difference was higher for private than public hospitals. The average number of claims per hospital was lower in Patiala because of the lower level of enrolment accompanied by a lower utilization rate. It might be speculated that such circumstances may have led providers to inflate the claim amount to achieve a desired level of profit from the limited number of cases reimbursable through RSBY. In Yamunanagar, the average number of cases per hospital was relatively high; it is possible that meeting a profit target from the volume of the claims was perhaps easier, thus reducing the incentive to inflate claims.

Interestingly, the study detected little difference in service delivery (process of care) between public and private sectors. Thus, study results appear contrary to the general presumption (although contentious) that the private sector offers superior care compared to the public sector (Das and Hammer, 2007), though structural quality was indeed reported to be higher in private hospitals. We found that RSBY, despite its PPP design, was not able to ensure access to all the types of care that the scheme had intended, though it did provide access to the private sector. We cannot confirm or refute that the PPP model produced good quality care in all aspects.

RSBY participants reported slightly better satisfaction when compared to non-RSBY participants. There could be two possible explanations for this. Firstly, as the assessment of hospital quality of care was self-reported, RSBY beneficiaries who previously lacked access may have been grateful for the facilities provided to them, regardless of whether they met recognised quality standards. Similar observations have been made for Vietnam (Wagstaff, 2007). Indeed, RSBY beneficiaries reported greater satisfaction than non-RSBY participants for aspects of care that would not have differed based on source of payment (e.g. cleanliness). The second possible explanation is in line with findings of Devadasan et al., that the insurance scheme might have negotiated a higher quality of care for its members (Devadasan et al., 2011). However, we found no evidence that there was such negotiation.

The evidence of substantial patient expenditure (at times nearly equal to that of the uninsured) is consistent with findings from other countries with regard to health insurance schemes for the poor (Acharya et al., 2012). The RSBY insurance scheme, an example of a PPP, was designed to take advantage of provider pluralism, but it is important to note that giving access to the private sector induced higher patient costs than were incurred in public hospitals. Consistent with the findings from the National Sample Survey, most OOP expenditure was related to drugs and diagnostics (NSS, 2015), and patients' reported expenditures provided evidence of lower compliance of private hospitals with provision of free medicines and diagnostics. Unless this is monitored and controlled, enabling access to private facilities risks resulting in higher OOP expenditure than would otherwise be the case.

The study cannot provide a definitive answer as to whether RSBY as a PPP mechanism has been overwhelmingly effective in improving access to hospitalization for the poor. Among the study's limitations is that it is not possible to show the extent to which insurance firms took profits from the scheme. We also cannot demonstrate that RSBY was associated with significant patient expenditure with the rigour that would be obtained, for example, in an impact evaluation study. Although the current study showed that not all mandated services were being offered, it could not demonstrate whether or not RSBY beneficiaries could access all needed services. Many of the flaws in RSBY would be predicted from a PPP arrangement under a weak regulatory and administrative system, notably non-compliance with contract terms on provision of services including failure to provide all required services free-of-charge, as indicated by the fact that payment for services was not at all uncommon even with insurance.

## 5 Conclusions and recommendations

RSBY was based on a PPP model governed through a series of contracts which enabled the poor to access private health services, even if compliance with contractual terms was poor.

An OECD review that examined policies and institutions that underpin supervision of quality in healthcare, using Mexico as a case study, suggests regulatory authority needs to be strong and independent of ministerial power (OECD, 2016). Before implementing more comprehensive health insurance, Colombia extensively restructured its delivery system to impose standards for healthcare providers (Glassman et al., 2009). In contrast, there was no strong, best practice institutional framework to guide the RSBY implementation process. RSBY's PPP framework and implementing mechanisms lacked a sufficiently strong regulatory environment with implications of weak governance and monitoring processes and consequences for expansion of care, provider compliance and user satisfaction. The fact that private service providers participated in the RSBY scheme, and that the scheme grew very rapidly to cover about 150 million beneficiaries, indicates that in many ways it was a successful public service model. However, there were indeed weaknesses in coverage of the BPL population, accessibility and availability of care, and compliance with scheme rules. Some of these were inherent problems with the scheme, such as achieving high enrolment of beneficiaries within a voluntary scheme and ensuring accessibility of care when hospitals clustered in urban areas; others were likely results of weak regulation and monitoring, such as non-compliance with requirements on free care.

A number of recommendations can be made to seek to seek to tackle the weaknesses.

• Greater supervision of both enrolment and empanelment should be provided by government. A conscious effort needs to be made to empanel more hospitals while ensuring quality of services.
• Engaging the private sector brought advantages, but stricter monitoring is required of all hospitals providing services.
• Regular medical and social audits of providers should be conducted, and sanctions imposed on providers who do not comply with contract terms. Ensuring that users are not exposed to cash payments for services which should have been free is an imperative.
• Greater modification of contracts at state, and if necessary, district level should be encouraged to meet the requirements of local conditions; this may require states to commit additional administrative manpower and support further capacity development.
• A strong monitoring and evaluation framework and plan needs to be incorporated in the contract design with a separate budget and dedicated human resources. Third party monitoring could be helpful and might bring significant improvements.
• Further thought needs to be given to the appropriate allocation of responsibility within the state government.

All these recommendations are relevant not just to RSBY, but also to the PPP framework adopted in Ayushman Bharat Yojana into which RSBY has been subsumed.

## Financial support and sponsorship

This work was supported by a Wellcome Trust Capacity Strengthening Strategic Award to the Public Health Foundation of India and a consortium of UK universities.

## Figures and Tables

**Table A1:** Proportion of enrolled beneficiaries in RSBY Scheme in Patiala district^a^.

		Under-5	5–14 yrs	15–24 yrs	25–44 yrs	45–64 yrs	>64 yrs	Total
Total population	Male	1,101,207	188,359	187,462	299,791	164,773	62,894	2,004,486
	Female	975,969	147,421	161,995	283,727	158,504	58,282	1,785,898
Eligible population (BPL)	Male	9,200	50,013	18,785	26,349	11,787	6,219	122,353
	Female	9,400	49,880	18,802	26,420	11,758	6,227	122,487
Enrolled population	Male	184	3,601	3,156	6,403	4,102	1,984	19,430
	Female	235	2,494	3,140	7,292	4,292	1,395	18,848
% enrolled of eligible population	Male	2.0%	7.2%	16.8%	24.3%	34.8%	31.9%	0.159
	Female	2.5%	5.0%	16.7%	27.6%	36.5%	22.4%	0.154
	All	2.25%	6.10%	16.75%	25.95%	35.65%	27.15%	0.156

a Similar data for Yamunanagar district were not available.

**Table A2:** Reimbursed and Claimed amounts.

District →	Reimbursed		Claimed	
	Patiala	Yamunanagar^a^	Patiala	Yamunanagar^a^
No of claims	992	5,903	992	5,903
Mean value (INR)	4,210	5,138	8,344	5,140
Std. deviation	3,992	4,175	6,520	4,175
P value	<0.01		<0.01	

Mean amount reimbursed by the insurance company to the hospital (private vs public)						
Hospital type →	Patiala		Yamunanagar		Both districts consolidated	
	Private	Public	Private	Public	Private^b^	Public^c^
No of claims	669	323	5,538	365	6,207	688
Mean value (INR)	3,708	5,250	5,140	5,105	4,985	5,173
SD	3,793	4,193	4,164	4,339	4,150	4,269
Difference	<0.01		<0.01		0.39	
**Mean amount claimed from the insurance company by the hospitals (Private vs public)**						
**Hospital type →**	**Patiala**		**Yamunanagar**		**Both districts consolidated**	
	**Private**	**Public**	**Private**	**Public**	**Private**	**Public**
No of claims	669	323	5,538	365	6,207	688
Mean value (INR)	8,159	8,724	5,143	5,105	5,468	6,804
SD	6,532	6,487	4,165	4,339	4,577	5,742
P Value	0.287		<0.01		<0.01	

a 140 claims were rejected in Yamunanagar district.

b 120 claims from private hospitals were rejected.

c 20 claims from public hospitals were rejected.
